# Supplementary material for: Demonstration of resonant tunneling effects in metal-double-insulator-metal (MI2M) diodes
Source: Nat Commun. 2021 May 18;12:2925. doi: 10.1038/s41467-021-23182-0 (PMC8131372; doi:10.1038/s41467-021-23182-0)
Supplement: Supplementary file 1 — Supplementary Information [file 41467_2021_23182_MOESM1_ESM.pdf]

Demonstration of resonant tunneling effects in metal-double-insulator-metal (MI^2M) diodes - Supplementary Information

Amina Belkadi

Department of Electrical, Computer and Energy Engineering,
University of Colorado at Boulder, Boulder, CO 80309-0425, USA. (email: amina.belkadi@colorado.edu)

Ayendra Weerakkody

Department of Electrical, Computer and Energy Engineering,
University of Colorado at Boulder, Boulder, CO 80309-0425, USA. (email: ayendra.weerakkody@gmail.com)

Garret Moddel*

Department of Electrical, Computer and Energy Engineering,
University of Colorado at Boulder, Boulder, CO 80309-0425, USA. (email: moddel@colorado.edu)

Simulation-based resonant tunneling analysis - transmission probability

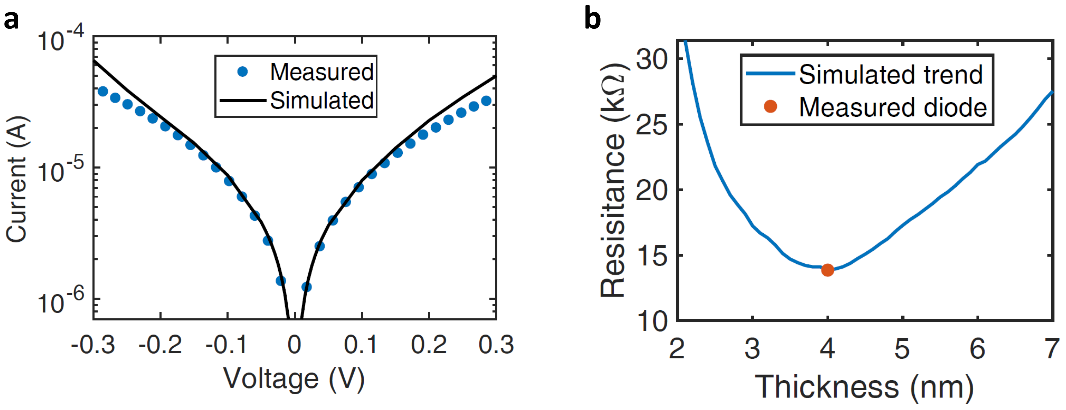

Figure 1: **Ni/NiO/Al_2O_3/Cr/Au diode simulation analysis.** (a) Measured (blue filled circles) and simulated (solid black line) $I(V)$ characteristics for 4 nm NiO Ni/NiO/Al_2O_3/Cr/Au diode. (b) Simulated resistance vs. NiO thickness trend (solid blue line) with the measured 4nm NiO Ni/NiO/Al_2O_3/Cr/Au diode (orange filled circle) used in the fit. The figure shows a drop in resistance with the increase in thickness between 2 and 4 nm.

Fig. 1b shows the relationship between zero-bias resistance and NiO thickness, by varying the thickness of NiO from 2 to 7 nm, using simulation fitting parameters of the measured 4 nm structure from Fig. 1a (represented by an orange filled circle). The results show that contrary to the expected increase in resistance with thickness, a drop in resistance in observed from 2 to 4 nm.

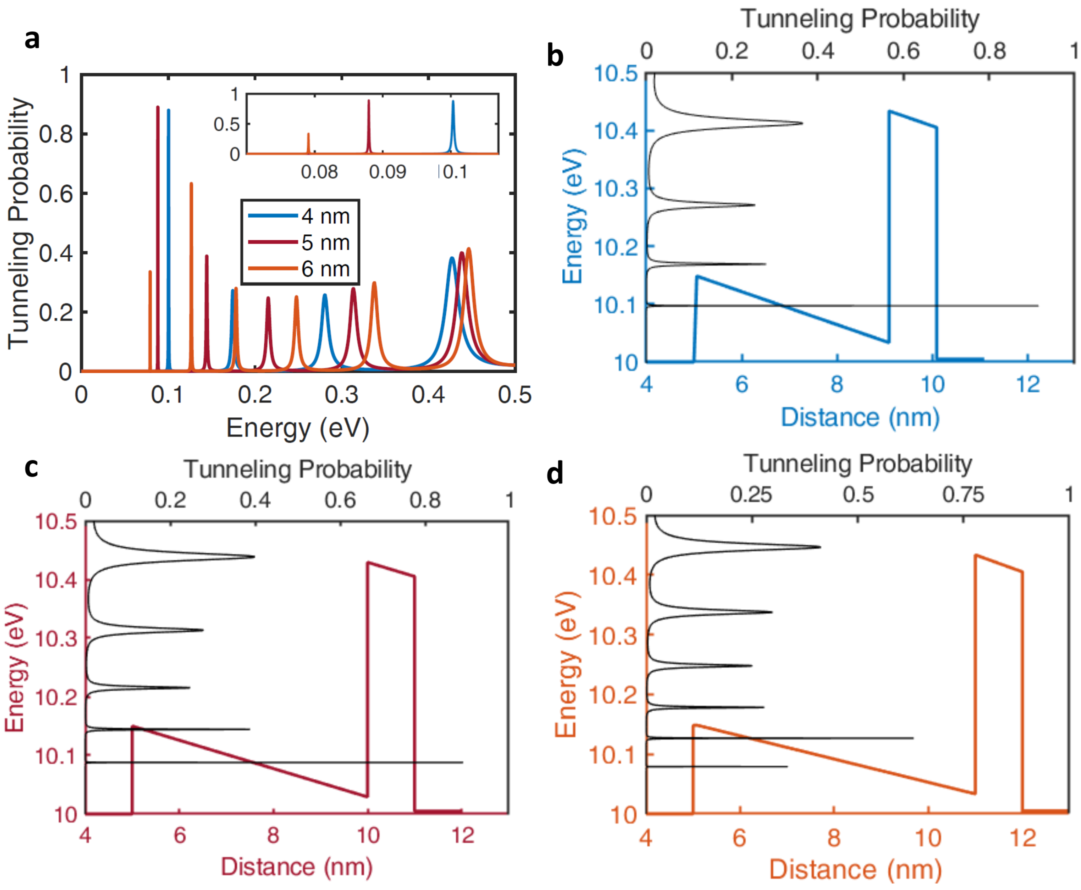

Figure 2: (a) Tunneling probability as a function of energy of simulated 4, 5 and 6 nm strcutures using the quantum mechanical tunneling simulator. The inset presents a close-up on tunneling probability of these diodes around an energy of 0.1 eV. Energy band diagrams and tunneling probability of (b) 4 nm, (c) 5 nm and (d) 6 nm structures.

Fig. 2 shows the tunneling probability as a function of energy for 4 nm and 6 nm thick NiO in Ni/NiO/Al_2O_3/Cr/Au

simulated structures. The simulator uses a transfer matrix method to solve a time-independent Schr$o$dinger equation and calculate transmission amplitudes, with a Hamiltonian matrix constructed to determine the bound states in a quantum well [1]. Transmittance is defined as the tunneling probability of electrons multiplied by the Fermi-Dirac distribution. Tunneling probability $T(E)$ is the ratio of outgoing to incoming quantum mechanical probability current density. $T(E)$ is the result of electron wave function interference with itself in the quantum well through reflections. In off-resonance, this interference is destructive and $T(E)$ is very small. At energies that align with the quantum well's quasi-bound states, the interference is constructive which results in the cancellation of reflected waves and the enhancement of transmitted one. Thus, resonant tunneling occurs when the majority of electrons tunnel with a $T(E)$ that sharply peaks to unity at these specific energies. The 4 nm structures $T(E)$ peaks close to 0.1 eV, the 5 nm close to 0.09 eV and the 6 nm close to 0.13 eV as shown in Fig. 2b, 2c and 2d. All of the three simulated thicknesses demonstrate sharp tunneling probability maxima around 0.1 eV, where a quasi-bound state with a narrow energetic bandwidth exists, through which electrons can tunnel. If these sharp peaks were identical, we would expect the 5 nm structure to exhibit a lower resistance compared to the 4 nm structure since its tunneling probability peaks with a relatively higher amplitude and closer to the Fermi-level, where more electrons exist due to the Fermi-Dirac distribution. This is not the case here as each of these peaks has a different lifetimes needed for electrons to decay out of the quasi-bound state. The lifetime associated with any of these states is related to the tunneling probability near resonance and to the energy width of the resonance by the uncertainty principle [2]. Both the Fermi-Dirac distribution of electrons and the energy width of the resonance peak must be taken into account when determining the current density. The 5 nm structure has a longer lifetime, a higher tunneling probability and a narrower resonance energy width. The 4 nm structure, with its wider bandwidth and a higher energy (0.01 eV above the 5 nm structure as depicted in the insert of Fig. 2a), results in a higher current density and lower resistance since more electrons tunnel through the quasi-bound state compared to the 5 nm structure. This is why the lowest resistance is observed at 4 nm in Fig. 1b. Because the quasi-bound state energy level does not have a large percentage of electrons tunneling through it, since there are not enough electrons at that level at room temperature, we do not observe the expected negative differential resistance that occurs when tunneling through a resonant state. Instead, we observe a reduction in resistance as a small fraction of electrons tunnel through that energy level. Observing a negative differential resistance in this structure would require a tighter electron Fermi-Dirac distribution around the quasi-bound state energy level, which can be achieved only at lower temperatures. For these reasons, we refer to the observed phenomena as near-resonant tunneling effects as opposed to pure resonant tunneling.

Material parameters used in simulation

Table 1: Work function (Φ) values used in simulations versus those reported in literature.

Material	Fitted values	Literature values	References
Ni	$\Phi = 5$ eV	$\Phi_{Ni} = 5.01$ eV	[3]
Cr/Au	$\Phi = 4.47$ eV	$\Phi_{Cr} = 4.5$ eV	[4]
		$\Phi_{Au} = 5.1$ eV	[3]

Band diagrams computation

Resistive voltage division

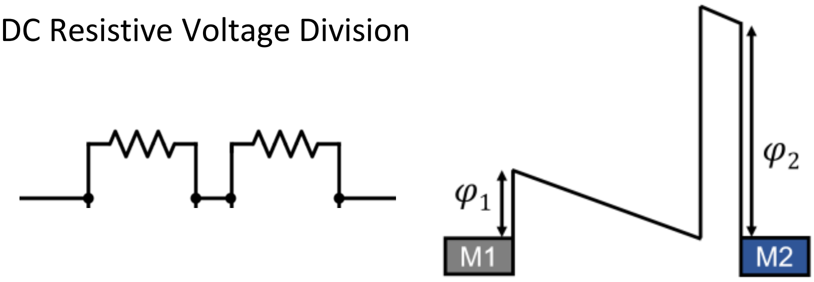

Figure 3: DC resistive voltage division simplified circuit model and corresponding band diagram.

To determine the energy-band profile at a certain voltage bias (V_D) at DC, we first determine the resistive voltage division across each insulators. Fig. 3 presents the simplified circuit diagram of the diode stack at DC as two resistors in series. The barrier heights (ϕ) are determined as

$$\phi = WF - EA \tag{1}$$

where WF is the metal's work function and EA is the oxide electron affinity. The voltage drop across each insulator is then determined as

$$\Delta V_j = (V_D - V_{bi}) \times (\%V_{RD}) \tag{2}$$

where $V_{bi} = \phi_1 - \phi_2$ is the built-in potential and $\%V_{RD}$ is the percentage voltage drop due to resistive voltage division. This is a fictitious resistance designed to fit the DC $I(V)$. The voltage drop (ΔV_j) represents the slope of the conduction band of the jth insulator. Drawing the band diagram becomes a simple matter of

$$E_{BD} = E_F + \phi_1 - \sum_{j=1}^{N} \frac{\Delta V_j \times x}{t_j} \tag{3}$$

where E_{BD} is the energy band diagram vector in eV and x is the displacement vector and t_j is the thickness of the jth oxide layer. For this particular structure, the thin Al_2O_3 layer results in a lower applied voltage (17%) compared to the 83% applied across the thicker 3.5 nm of NiO, as illustrated in Fig. 3. The two competing effects of the thin and high barrier in Al_2O_3 result in similar slope band bending as the lower barrier, thicker NiO insulator. Even though tunneling in an MI^{2}M diode goes through the entire structure and cannot be physically divided into two resistance values that sum to the total resistance, we were able to use this fictitious resistance method to achieve accurate fits of DC $I(V)$ characteristics.

Capacitive voltage division

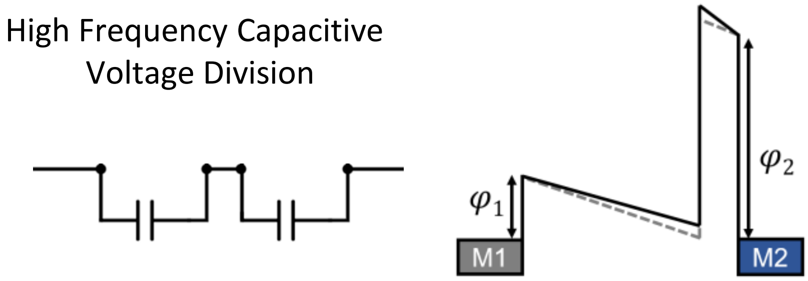

Figure 4: Capacitive voltage division circuit model and corresponding band diagram. The dashed lines represent the resistive voltage division band diagram for comparison.

For frequencies well above cutoff, voltage division is determined by the oxide capacitors, as seen in Fig. 4. The voltage drop across each insulator becomes

$$\Delta V_j = (V_D - V_{bi}) \times \frac{x_j/\epsilon_j}{\sum x_j/\epsilon_j} \tag{4}$$

where x_j and ϵ_j represent the thickness and dielectric constant, respectively, of the jth layer. The percentage voltage drop due to capacitive voltage division (V_{CD}) is represented here as $(x_j/\epsilon_j)/(\sum x_j/\epsilon_j)$. This comes from the voltage division across two capacitors in series where

$$C_{Total} = \frac{C_1 C_2}{C_1 + C_2} \tag{5}$$

For capacitive voltage division in the NiO/Al_2O_3 structure, the lower Al_2O_3 high frequency dielectric constant results in more voltage applied across Al_2O_3 and more bending, as seen in Fig. 4. Similarly, the higher NiO dielectric constant results in lower voltage applied and thus, less bending compared to the resistive voltage division.

Thickness dependent material properties The measured 4 nm and 5 nm NiO diodes exhibited lower and higher resistance than expected from simulations, respectively. One possible explanation is thickness dependent

material properties, where NiO chemical compositions varies with thickness [5]. To check for this, we performed x-ray photoelectron spectroscopy (XPS) measurements of 20 nm NiO and 2 nm NiO deposited on 20 nm Al_2O_3. We found that the Ni 2p XPS spectra shoulder peak (855.42 eV) of the 2 nm NiO sample is more pronounced than the NiO main peak (854.19 eV). This shoulder comes mostly from the pyramidally coordinated Ni atoms at the surface of these nano-structured systems [5]. Another contributing factor to the formation of a shoulder in Ni 2p spectra of thin NiO is that when the thickness of NiO in the order of a few monolayers, growth of NiO happens through small islands with determined heights ascribed to the formation of many nucleation centers at the grain boundaries of the polycrystalline materials [5]. When NiO thickness is varied, the shoulder peak intensity of Ni 2p spectra varies, implying thickness dependent NiO island formation. The spread in resistance values is thus attributed to a combination of thickness sensitive interfacial layer properties [6, 7] and thickness dependent NiO chemical composition.

Calculation of coupling efficiency The coupling efficiency η_c presents the ratio of AC power delivered to the diode to the power absorbed by the antenna. The simplified coupling efficiency between a diode and an antenna, for a diode without capacitance, can be calculated as

$$\eta_c^{simplified} = \frac{4R_A R_D}{(R_A + R_D)^2} \tag{6}$$

where R_A is antenna impedance and R_D is diode impedance. For energy harvesting applications where MIM diodes operate as rectifiers, the diode capacitance (C_D) and resistance (R_D) determine the maximum operation frequency $f_c = 1/(2\pi RC)$. This modifies the coupling efficiency to include frequency-dependent diode capacitance (C) such that

$$\eta_c = \frac{4R_A R_D}{(R_A + R_D)^2 + (R_A R_D \omega C_D)^2} \tag{7}$$

This equation assumes the antenna reactance is negligible compared to the diode reactance, which is not always the case at high frequency (> 1 terahertz) and can reduce the coupling efficiency by a factor of 10 or more.

References

[1] Sachit Grover and Garret Moddel. Engineering the current–voltage characteristics of metal–insulator–metal diodes using double-insulator tunnel barriers. Solid-State Electronics, 67(1):94–99, 2012.

[2] Emilio Eugenio Mendez and Klaus von Klitzing. Physics and applications of quantum wells and superlattices, volume 170. Springer Science & Business Media, 2012.

[3] David R Lide CRC handbook of chemistry and physics. CRC press, 85, 2004.

[4] Josef Hölzl and Franz K Schulte. Solid surface physics. Springer, 1–150, 1979.

[5] Erin L Ratcliff, Jens Meyer, K Xerxes Steirer, Andres Garcia, Joseph J Berry, David S Ginley, Dana C Olson, Antoine Kahn, and Neal R Armstrong. Evidence for near-surface niooh species in solution-processed nio x selective interlayer materials: impact on energetics and the performance of polymer bulk heterojunction photovoltaics. Chemistry of Materials, 23(22):4988–5000, 2011.

[6] Don Ayendra Dilshan Chathuranga. Engineered High-k Oxides. PhD thesis, University of Liverpool, 2016.

[7] Ying-Ta Shih, Chien-Yu Su, Chung-Wei Tsai, and Wei Pan. Thickness and ordering temperature of surface nio/ni systems. AIP Advances, 4(2):027117, 2014.
